# Phylogenetic Diversity in Core Region of Hepatitis C Virus Genotype 1a as a Factor Associated with Fibrosis Severity in HIV-1-Coinfected Patients

**DOI:** 10.1155/2017/1728456

**Published:** 2017-11-12

**Authors:** Micaela Parra, Natalia Laufer, Julieta M. Manrique, Leandro R. Jones, Jorge Quarleri

**Affiliations:** ^1^Instituto de Investigaciones Biomédicas en Retrovirus y Sida (INBIRS), Facultad de Medicina, Universidad de Buenos Aires, Paraguay 2155, Piso 11, C1121ABG Buenos Aires, Argentina; ^2^Laboratorio de Virología y Genética Molecular (LVGM), Facultad de Ciencias Naturales y Ciencias de la Salud Sede Trelew, Universidad Nacional de la Patagonia San Juan Bosco, 9 de Julio y Belgrano S/N, Chubut, 9100 Trelew, Argentina; ^3^Consejo Nacional de Investigaciones Científicas y Técnicas, Av. Rivadavia 1917, C1083ACA Buenos Aires, Argentina

## Abstract

High hepatitis C virus (HCV) genetic diversity impacts infectivity/pathogenicity, influencing chronic liver disease progression associated with fibrosis degrees and hepatocellular carcinoma. HCV core protein is crucial in cell-growth regulation and host-gene expression. Liver fibrosis is accelerated by unknown mechanisms in human immunodeficiency virus-1- (HIV-1-) coinfected individuals. We aimed to study whether well-defined HCV-1a core polymorphisms and genetic heterogeneity are related to fibrosis in a highly homogeneous group of interferon-treated HIV-HCV-coinfected patients. Genetic heterogeneity was weighed by Faith's phylogenetic diversity (PD), which has been little studied in HCV. Eighteen HCV/HIV-coinfected patients presenting different liver fibrosis stages before anti-HCV treatment-initiation were recruited. Sampling at baseline and during and after treatment was performed up to 72 weeks. At inter/intrahost level, HCV-1a populations were studied using molecular cloning and Sanger sequencing. Over 400 complete HCV-1a core sequences encompassing 573 positions of C were obtained. Amino acid substitutions found previously at positions 70 and 91 of HCV-1b core region were not observed. However, HCV genetic heterogeneity was higher in mild than in severe fibrosis cases. These results suggest a potential utility of PD as a virus-related factor associated with chronic hepatitis C progression. These observations should be reassessed in larger cohorts to corroborate our findings and assess other potential covariates.

## 1. Introduction

Hepatitis C virus (HCV) is an enveloped, positive-stranded RNA virus belonging to the genus* Hepacivirus* in the family Flaviviridae. HCV infection is the major cause of chronic liver disease. Total global viraemic HCV infections were estimated in 80 (64–103) million infections [1]. Of them, more than 4 million are coinfected with the human immunodeficiency virus (HIV) as both viruses share common transmission routes [2].

HCV-chronic infection is responsible for different hepatic damage including oxidative stress, insulin resistance, steatosis, fibrosis, apoptosis, and hepatocellular carcinoma (HCC) [3, 4]. Such alterations are attributed to changes in gene expression patterns in the liver due to virus replication dynamics [5].

Assessment of liver fibrosis has important implications for prognosis and for decision-making on the onset of therapeutic approaches. Liver fibrosis is the excessive accumulation of extracellular matrix proteins as a consequence of chronic aggression on the tissue. In the natural course of HCV infection, advanced liver fibrosis results in cirrhosis and concomitantly in HCC [6]. However, there are extrinsic factors as many as individual-inherent factors that could lead to fibrosis progression. It has been shown that fibrosis progression significantly varies among HCV-infected patients. Some of them evolve rapidly into several stages while others evolve into mild fibrosis that could not even progress at all. Some host factors, such as male gender and age at the time of infection, are the two main determinants in the natural course of HCV infection [7]. HIV-related immunosuppression is associated with accelerated liver disease in individuals with HIV/HCV coinfection [8].

HCV genetic variability brings challenges when making generalizations about the aggressiveness of different genotypes and the response to antiviral therapy. Low fidelity RNA-dependent RNA polymerase contributes to increasing mutational rate, generating a collection of closely related genomic variants called* quasispecies* [9].

Several studies have demonstrated that the highly conserved HCV core protein plays an important role in the pathogenesis and progression of the disease due to its ability to interact with a wide spectrum of viral and cellular proteins, including protooncogenes [10]. Particularly, amino acid substitutions at positions 70 and 91 in the HCV core region have been identified as predictors of hepatocellular carcinoma among genotype 1b-infected patients from Japan [11–13]. Hence, these polymorphisms might be considered surrogate markers for hepatic disease stages or the eventual development of HCC. However, these mutations have not been assessed in HCV genotype 1a/HIV coinfected patients. Instead other authors have reported the use of other noninvasive test indexes (Forns, APRI, and FIB-4) based on a panel of standard laboratory markers [14, 15] as well as an alternative statistical approach based on generalized linear models (GLMs), to identify factors associated with liver disease progression [16, 17].

In the present longitudinal study we report the characterization of HCV genotype 1a core sequences* in vivo* in HIV coinfected patients and its relationship with hepatic damage. Besides standard analysis including quasispecies heterogeneity, intrahost positive selection pressure, and changes at codons 70 and 91, we analyzed the viral Faith's phylogenetic diversity* (PD)*, which has received little attention in HCV and viruses in general.

## 2. Material and Methods

### 2.1. Patients

Plasma samples longitudinally collected from eighteen HIV positive patients coinfected with HCV genotype (Gt) 1a were analyzed. For each patient, sampling times include before, during, and at end of interferon-based therapy. Clinical records were reviewed, clinical and laboratory data including CD4^+^ T cell count (flow cytometry double platform, BD FACS Canto; BD Biosciences, San Diego/California, USA) and plasma HCV viral load (VL) by a quantitative reverse transcription- (RT-) PCR assay (COBAS AMPLICOR HCV MONITOR version 2.0; lower limit of quantification of 2.78 log_10_ IU/ml; Roche Molecular Systems). These parameters were recorded for each sample from each patient during the entire follow-up (Table 1). Patients were treated with anti-HCV pegylated-interferon (peg-IFN) plus ribavirin- (RBV-) based therapy (the standard of care when patients were recruited). They were all on HAART with undetectable HIV-1 RNA (<50 copies/mL).

Liver fibrosis was evaluated by liver biopsy within 12 months before HCV treatment initiation. Results were reported using METAVIR scoring system [18]. Patients were selected for low or high fibrosis scores. Ten patients had absent-to-moderate fibrosis (METAVIR F0–F2), while eight had severe fibrosis-to-cirrhosis (METAVIR F3-F4). Regarding anti-HCV therapy response, three patients were responders (R) (defined as undetectable HCV RNA 24 weeks after treatment completion), and 15 were nonresponders (NR) [19]. Based on the observed scores, patients were categorized as having no or moderate fibrosis (*low* fibrosis grade; METAVIR scores F0–F2), or severe fibrosis-to-cirrhosis (*high* fibrosis grade; METAVIR scores F3-F4) [20, 21].

Samples were collected during the course of routine clinical follow-up and stored at −80°C until use. For each patient, we tested plasma samples collected before anti-HCV therapy initiation and then, longitudinally across at least 12 weeks and for a maximum of 72 weeks after antiviral therapy initiation.

### 2.2. Amplification, Cloning, and Sequencing of HCV Core Gene

Viral RNA was isolated from plasma samples presenting VL higher than 2.78 log_10_ UI/mL using PureLink® Viral RNA/DNA Mini Kit following the manufacturer's instructions (Invitrogen, Life Technologies, USA). The complete HCV core encoding sequence was amplified using primers core_EF (5′-CGAAAGGCCTTGTGGTACTG-3′, position 272–291) and Core_ER (5′-CTCCCGAACGCAGGGCAC-3′, position 1020–1037), and then primers Core_IF (5′-TTGTGGTACTGCCTGATAGGGT-3′, position 281–302) and Core_IR (5′-ATGCTTGAGTTGGAGCAGTCG-3′, position 956–976) in a nested reverse transcriptase polymerase chain reaction (RT-PCR) generating a 695 bp amplicon. The RT program included incubation at 25°C for 5 min and 60 min at 37°C followed by RT enzyme (Moloney Murine Leukemia Virus Reverse Transcriptase, Thermo Fisher Scientific) deactivation at 94°C for 15 min. The first PCR program included 40 cycles each of 30 s at 94°C, 30 s at 48°C, and 1 min at 72°C using a Taq DNA polymerase (PB-L, Argentina), followed by a final extension at 72°C for 5 min. The PCR product was used in the nested PCR and the program included 25 cycles of 30 s at 94°C, 30 s at 48°C, 1 min at 72°C, and a final extension of 72°C for 5 min. PCR products were resolved by electrophoresis on 1% agarose gels.

At intrahost level, the HCV core sequence analysis involved the purification and cloning of those amplicons obtained at two most distantly separated sampling times including baseline with the pGEM®-T Easy Vector System (Promega) according to the manufacturer's instructions. The constructs generated were used to transform DH5*α Escherichia coli* competent cells. Thirteen to twenty white colonies were screened by colony PCR for each of the 31 samples for sequence analysis. Plasmid inserts were amplified using pGEM®-T-specific primers T7 and SP6 and subjected to double stranded sequence analysis with dye-labeled dideoxy terminators with both T7 and SP6 primers (genetic analyzer 3500xL, Applied Biosystems®, Life Technologies, USA). The core sequences from the two sampling time points selected (or only baseline in the case of the nonresponder patient E or responder patients) were edited and assembled using Sequencher software v.5.0.1 (Gene Codes Corporation, Ann Arbor, MI, USA).

Though PCR mutations are a well-known problem, our pipeline for clonal analysis included amplifications that were performed using sufficient molecules as template in order to minimize the error rate due to PCR.

### 2.3. Phylogenetic Analysis

Sequence alignments were obtained with the MAFFT program implementing iterative refinement, weighted sum-of-pair and scores, and a consistency score obtained from local alignments [22].

Phylogenetic trees were inferred by FastTree-2 [23], under the GTR + CAT model. FastTree automatically scales the tree searching effort proportionally to the number of sequences in the dataset [i.e., the program performs up to 4 × log⁡2(*N*) rounds of minimum-evolution nearest neighbor interchange (NNI), 2 rounds of subtree pruning regrafting (SPR) moves, and up to 2 × log⁡(*N*) rounds of maximum likelihood NNIs, where *N* is the number of unique sequences in the dataset]. We set the program to start from an exhaustive-search BIONJ tree and turned off top-hit heuristics to tree improve searches. Bootstrapping was used to both obtain branch supports and cope with phylogenetic uncertainty in evolutionary analyses. To this aim, we generated 1000 bootstrapped datasets using* Seqboot* [24] and analyzed the obtained data with FastTree. Phylogenetic diversity measures were obtained from each of these 1000 bootstrapped trees. Branch supports were computed by the* CompareToBootstrap.pl* script (distributed with FastTree). Trees were visualized and annotated in Dendroscope 3 [25].

### 2.4. HCV Quasispecies Heterogeneity Analyses

Sequence logos representing DNA and protein sequence alignments were produced using Skylign web server http://skylign.org [26].

Nucleotide sequences were analyzed to assess both inter- and intrapatient quasispecies heterogeneity dynamics during the follow-up. Sequences corresponding to different molecular species were classified as haplotypes. Viral diversity was evaluated from phylogenetic trees, that is, phylogenetic diversity* (PD)* of Faith which reflects the total length of branches connecting haplotypes along the phylogenetic tree [27]. These analyses were performed using the PD function in the Picante package [28]. Given that diversity indices can be biased by differences in sequence effort, we used a normalized* PD (n-PD)*, which was calculated from (equally sized) subsets of sequences randomly drawn from the original pool of sequences from each sample [29].

### 2.5. Intrahost Evaluation of HCV Core Variability and Positive Selection Pressure

In order to evaluate HCV core variability at each amino acid position was calculated as the entropy, defined as –Σ*P*(*si*)log⁡*P*(*si*), where *P*(*si*) is the probability of a given amino acid (*s*) appearing at a given position (*i*). Entropy was computed and the consensus amino acid sequence was determined using the Entropy-ONE Web tool (https://www.hiv.lanl.gov/content/sequence/ENTROPY/entropy_one.html) [30].

Detection of selective pressure was performed using the methods of single likelihood ancestor counting (SLAC) [31], of fixed effects likelihood (FEL), and of mixed effects model of evolution (MEME) [32], as implemented in HyPhy v2.2.1 [33] with online Datamonkey software [34, 35]. The analysis was conducted with default options. *dN*/*dS* ratio greater than 1 and a *p* value less than 0.05 was considered to be positively selected. For a higher confidence in the inference of positive selection, only those sites exhibiting coincident results with at least two methods were defined as positively selected sites.

### 2.6. Statistical Analysis

Quasispecies diversity differences between sampling times in a single patient were calculated by paired-samples Wilcoxon test. Correlations between clinical and quasispecies diversity were evaluated by a regression linear model. All of these were implemented in R v.3.2.3. A *p* value 0.05 was considered significant.

## 3. Results

### 3.1. Nucleotide and Amino Acid Variability in Core Region of HCV-1a

**Figure 1:**
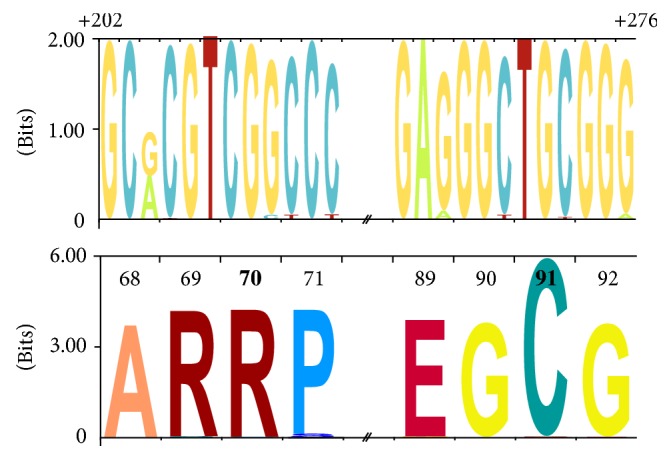
*Graphical representation of HCV core nucleotide and amino acid sequences from 335 haplotypes*. These were obtained from 18 HCV/HIV coinfected patients. The total height of each stack corresponds to a measure of the conservation of the column (the information content or entropy of that position expressed in bits). The relative height of each letter within a stack depends on the frequency of that letter at that position. Amino acid positions 70 and 91 are highlighted in bold.

A total of 407 HCV core gene molecular clones were sequenced from samples obtained before, during, and after anti-HCV peg-IFN + RBV treatment. These sequences corresponded to 335 unique variants (hereinafter* haplotypes*). All 335 haplotypes were quite similar; they showed a number of nucleotide changes but were mainly synonymous mutations (Figure 1). Since it is known that there are some relevant core amino acid substitutions associated with increased HCC risk and variable response to peg-IFN + RBV treatment in HCV-1b, we searched for those changes in our sequences from core HCV-1a isolates. Reported core mutations R70Q and L/C91M were not found. Nevertheless, two HCV-1a haplotypes exhibited two mutations at core (R70P and C91R); we did not find any characteristic mutations of fibrosis. Among 18 patients, any amino acid substitution over time was associated with anti-HCV therapy response.

### 3.2. Phylogenetic Analysis

**Figure 2:**
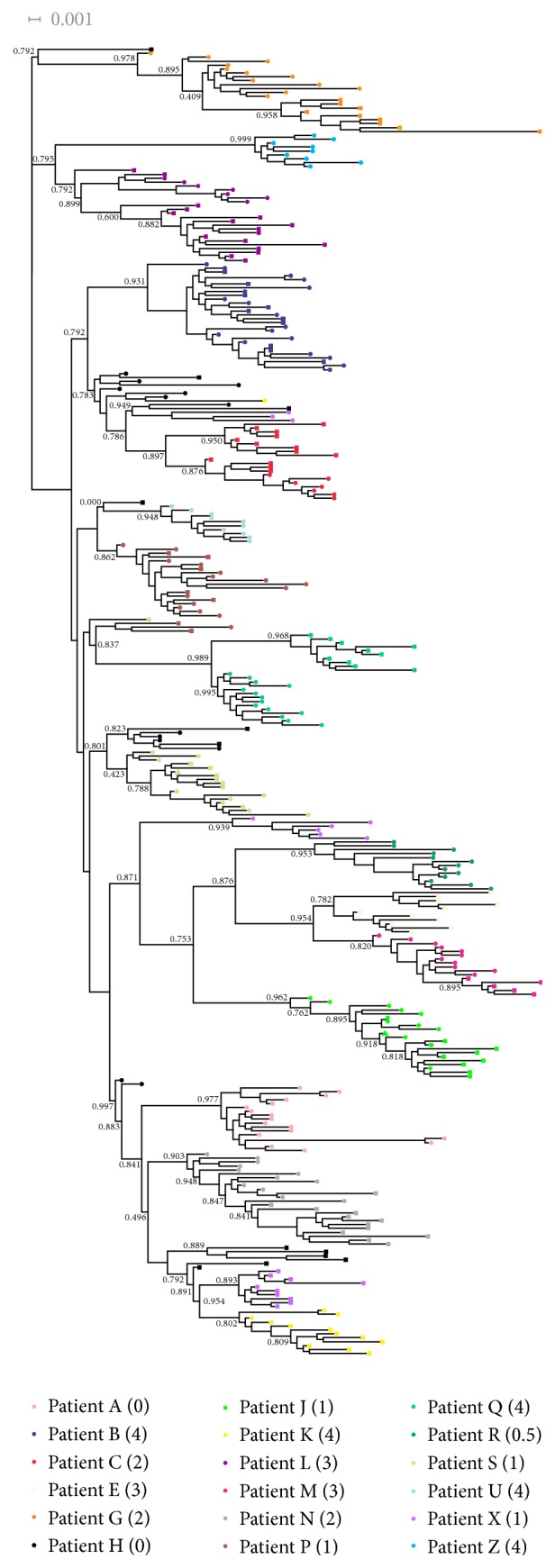
*Maximum likelihood phylogenetic midpoint routed tree of 335 HCV haplotypes from patients included in the study*. Patient-related clusters are identified in different colors with corresponding fibrosis stage between parentheses, as indicated at the top of the figure. Sampling times are coded by symbols (pretreatment: circles; intra- or posttreatment: squares; we coded haplotypes present in both sampling times as triangles). Numbers on branches correspond to relevant bootstrap values. Branch lengths are proportional to the number of nucleotide substitutions per aligned site (bar = 0.01).

All haplotypes characterized were patient-specific, meaning that there were no haplotypes shared between different individuals for the entire follow-up (Figure 2). In addition, each patient's haplotypes were highly related to each other compared to the haplotypes from other patients (Figure 2), in agreement with previous studies [36, 37]. The only exceptions were patients H and X (and in a lesser extent patient S), whose sequences were sparse across the phylogeny in the case of patient H and grouped in two different clades for patient X (Figure 2). As also reported previously, some patients presented temporally structured phylogenies, in which the haplotypes from the same sampling time were closely related in the tree (patients C, G, K, M, N, Q, and S; Figure 2), whereas others showed relatively dispersed phylogenetic patterns (patients B, J, L, and P).

### 3.3. Phylogenetic Diversity

**Figure 3:**
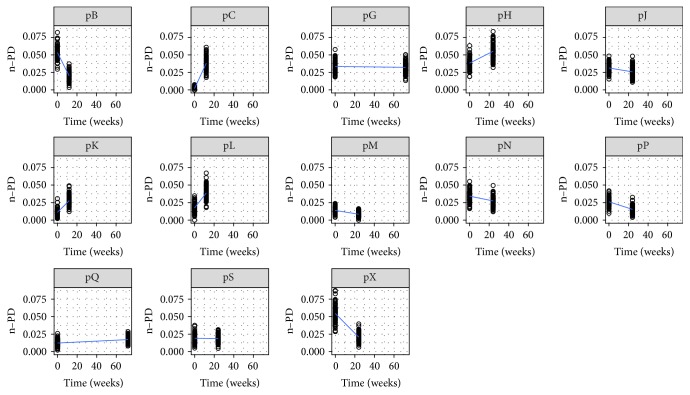
*Fluctuations in viral population phylogenetic diversity (n-PD)*. It was measured during the follow-up of nonresponders to IFN + RBV therapy HCV/HIV coinfected patients. Patients are identified by “p” (patient) letter followed by their letter code.

For intrapatient analyses, n-PD values were calculated from each sample and we compared phylogenetic diversity over time for each nonresponder patient with the exception of patient E because it was not possible to amplify some samples taken at least 12 weeks after therapy initiation. Differences between pretreatment and intra- (or post-) treatment samples were statistically significant for almost every nonresponder patients (Wilcoxon test, *p* < 0.01) with the exception of patients G and S (Wilcoxon test, *p* > 0.05). As depicted in Figure 3, viral diversity increased in patients C, H, K, L, and Q over time, whereas in patients B, J, M, N, P, and X, it decreased. We evaluated both correlations between these different fluctuations and CD4+ T lymphocytes total counts before therapy initiation or initial variability but no significant associations were found (simple linear regression, *p* > 0.05). Next, we separated nonresponder patients according to the evolution of viral diversity during treatment (i.e., an increase or a decrease) and we found that CD4+ T cell count pretreatment ranges overlapped (initial n-PD < final n-PD, CD4+ = [303–625] cells/mm3; initial n-PD > final n-PD, CD4+ = [262–817] cells/mm3).

### 3.4. Phylogenetic Diversity in Core Region of HCV-1a as a Factor Associated with Fibrosis Severity

**Figure 4:**
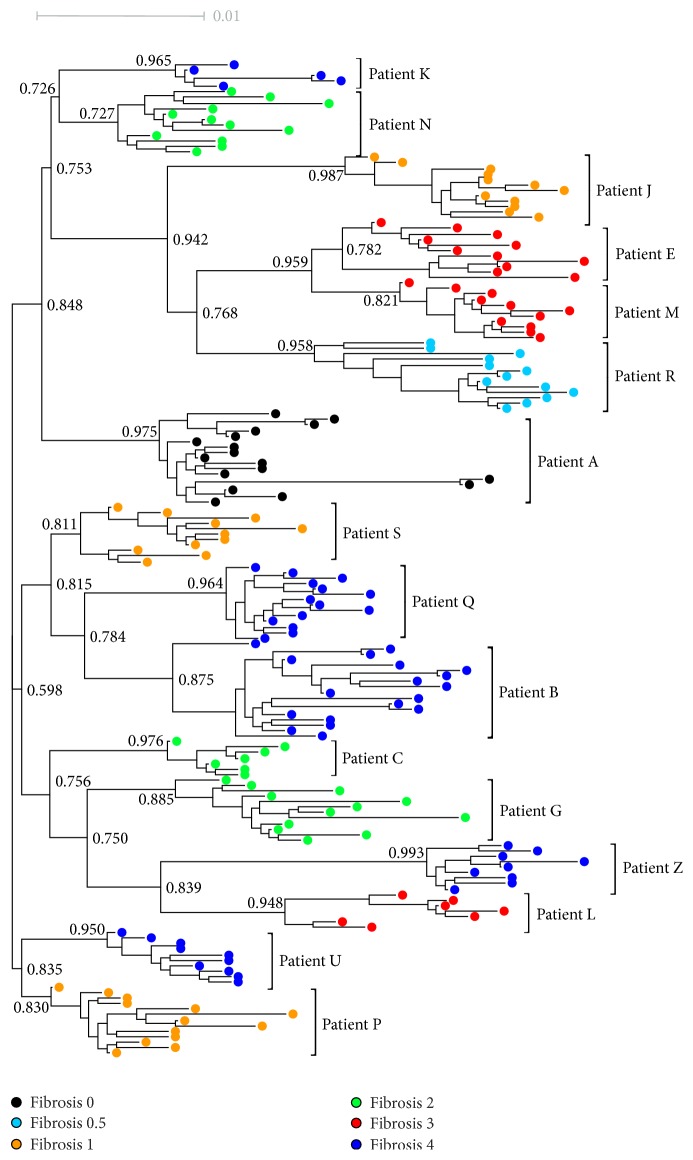
*Maximum likelihood phylogenetic midpoint routed tree of 182 HCV haplotypes isolated from baseline (pretreatment) samples*. Fibrosis score (measured within 12 weeks before IFN + RBV therapy initiation) is identified in different colors as indicated at the top of the figure. Haplotypes isolated from the same patient are indicated with patient letter next to the cluster. Some bootstrap values are given on branches. Branch lengths are proportional to the number of nucleotide substitutions per aligned site (bar = 0.01).

Due to conventional amino acid sequences, the analysis of core HCV-1a did not exhibit mutations associated with HCC risk as occurs for the same protein in HCV-1b. We analyzed phylogenetic relationships among HCV haplotypes from each sample obtained before therapy initiation and their relationship with fibrosis stages. Maximum Likelihood analyses recovered monophyletic groups from each patient with bootstrap values higher than 70% but no clustering according to the fibrosis stage was observed (Figure 4).

**Figure 5:**
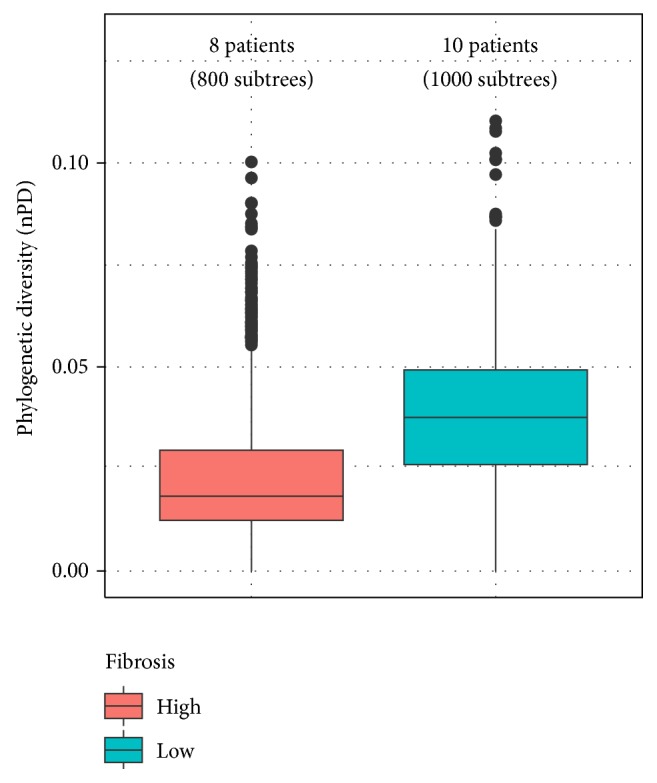
*Pretreatment HCV phylogenetic diversity (n-PD) categorized by fibrosis grade in HCV/HIV coinfected patients*. The boxplots represent the obtained results from 100 bootstrap trees. Lines over the boxes correspond to mean values, whereas boxes themselves indicate 1st and 3rd quartiles; whiskers show the maximum and minimum values, excluding the outliers values, which are represented by dots.

As mentioned above,* PD* has been little studied in HCV. Here, we assessed the relationship between a corrected* PD* measure (*n-PD*; Materials and Methods) and fibrosis grade* (FG)*. We calculated the virus* PDs* in all the studied patients and plotted the obtained results against the corresponding* FGs*. With* PD* being a phylogenetic index, it can be influenced by phylogenetic uncertainty, a frequently neglected but potentially important issue. Thus, to assess potential biases due to this phenomenon, we generated a sample of 1000 bootstrap trees and* PDs* were obtained from each of these trees.  Figure 5 displays boxplots corresponding to* PDs* obtained from several patients categorized by the corresponding* FGs*. Interestingly, the analysis revealed a negative relationship between the studied variables (*p* < 0.01), suggesting that the progression towards severe fibrosis stages may be associated with a decrease of* PD*.

A large number of studies have shown that there might be a relationship between CD4+ T cell counts and the severity of hepatic damage, but neither fibrosis nor viral diversity showed any association with CD4+ T cell total count among the studied patients (data not shown).

### 3.5. Intrahost Selection Pressure Analysis in Core Region

We analyzed concomitantly the selection pressure with three methods (SLAC, FEL, and MEME) from each patient and time sampling. As we had expected because of the high homogeneity in our sequences, we found only one site classified coincidently by FEL and MEME methods under positive selection in patient H (Table 2). No positively selected sites were reported by SLAC method.

## 4. Discussion

Core is the most highly conserved of all HCV proteins. Although core sequences are highly similar it is known that a few amino acid mutations can be associated with HCC risk and worse prognosis likely to changes in conformation and then function and protein stability. Many polymorphisms could alter predicted RNA motifs regulating oncogenic processes in a different way [38–40].

In terms of the variability in core alignments, we found both nucleotide and amino acid mutations with a variable prevalence in all patients and sampling times. However, most amino acid sequences were identical to the reference as reported elsewhere [41]. This high level of protein sequence conservation is expected since HCV core is associated with cellular pathways involved in the life cycle of the virus [42, 43].

Fibrosis progression in HCV-infected patients can evolve in HCC as a consequence of cirrhotic damage. Core protein has been marked as an important element in this process because it can interact with a wide variety of cellular proteins modulating cellular proliferation and apoptosis [44, 45]. Furthermore, the effects of genetic polymorphisms may interact with viral replication itself. R70Q and L/C91M mutations have been frequently associated with HCC risk and variable response to IFN + RBV therapy. However, we did not find any specific mutations from nonresponder patients or severe fibrosis-to-cirrhosis in this cohort of HCV 1a-coinfected patients. Furthermore, we did not find a positive selection for non-R70 variants during IFN + RBV therapy as reported before [46]. Majorly intrahost HCV evolution in our patients was characterized by a consistent negative selection over time, suggesting the increasing HCV adaptation to the host in chronic infection. This is in line with the recently reported analysis across all HCV genotypes, where codon positions appeared rarely identified to be positively selected and predominantly found to be under negative selective pressure, suggesting mainly a neutral evolution [47]. It seems clear that our finding is the result of the characteristics of patients selected for the present study who were infected with HCV-1a genotype, also observed in previous studies [48–50]. Despite the retrospective nature and the small sample size of our study, our results may suggest that R70Q and L/C91M key mutations are typical of HCV 1b-infected patients and they should not necessarily be associated with HCC risk or prognosis predictor in HCV 1a-infected individuals.

Interestingly, in our analysis we also observed that viral diversity before IFN + RBV combined therapy initiation was not associated with a nonresponder or a responder condition. In this sense, the results of the literature are controversial. While some authors argue that baseline diversity is higher in nonresponders, others argue that response to therapy is independent of initial diversity [51]. As observed in this study, diversity during follow-up of nonresponders varied without a fixed pattern. This supports the hypothesis that HCV evolution dynamics is patient-dependent [52, 53]. In this sense, there is evidence that genetic background of each study population should be considered in these analyses [54, 55]. An interesting example of the complexity of this interaction is offered by R70Q mutation. This substitution is associated with therapy failure in genotype 1b. However, it is the most common mutation in genotypes 5 and 6. HCV genotype 5 appears to be an easy-to-treat virus with response rates similar to those of genotypes 2 and 3 after a 48-week course of therapy. Response to treatment in patients with HCV genotype 6 may be at an intermediate level between the observed in genotype 1 and genotype 2 or 3 [56] but a limited number of patients have been studied till present.

We have observed synonymous point mutations in all haplotypes. Sometimes, the same nucleotide change was present in quasispecies isolated from different patients, but each individual showed haplotypes with a unique combination of mutations. A limited number of specific changes in defined positions of the gene could explain, in some way, different profibrotic potentials of core protein. Another possibility should not be ruled out: real profibrotic variants could exist and persist because of their greater fitness. Although a high number of population and clonal sequences were obtained, the conditions for enrolled patients (HCV genotype 1a and HIV coinfection) limited the number of patients in the present study. A power study is deserved to reach a stronger signal from the dataset in order to observe eventual amino acid substitutions in the core genetic region.

By phylogenetic analyses we have observed that the viral haplotypes associated with each fibrosis score did not seem to be related to each other but were clustered in a patient-dependent manner representing a virus-to-host adaptation of HCV in chronic infection and IFN-based therapy, as observed in a previous study of HCV 1b-infected patients [57]. However, a sparse HCV haplotypes distribution was observed in some cases (more prominently H and X) which may reflect eventual mixed infection (or superinfection) with different HCV-1a strains. Considering that HCV variants prevalent at different stages of infection may have specific immunological properties and that HCV-specific adaptive immune response may be impaired in HIV coinfected patients, the selection pressure on the pool complex of viral variants to achieve a stable adaptation to the host may not still occur in these patients.

Decreased HCV genetic heterogeneity could be interpreted both as a cause or a consequence of increased liver damage. In line with the hypothesis that considers the level of viral diversity as the cause of the tissue status several studies have suggested the idea that baseline diversity of HCV population is an indicator of hepatic disease progression including those performed in HCV-monoinfected [58, 59], HCV/HIV coinfected adults [60], and transplant patients chronically infected with HCV [61, 62]. Regarding the second hypothesis Harouaka et al. [63] argued that the architecture of tumoral hepatic tissue would be associated with restrictions in HCV replication. This could explain the low diversity in the quasispecies population present in more severe fibrosis stages. If this were the case, phylogenetic diversity could be a potential, noninvasive indicator of liver harm but further investigation is deserved.

In conclusion, we have observed no relationships between particular HCV-1a core polymorphisms and fibrosis severity or response to IFN + RBV therapy in HCV/HIV coinfected patients. On the other hand, we observed a significant relationship between fibrosis degree and HCV-1a core* PD*, which, in a multifactorial scenario, appears to be a relevant HCV-related factor involved in the progression of chronic hepatitis C. However, our results should be interpreted cautiously considering the low number of cases, potential selection bias, and the retrospective nature of the study. It deserves further investigation including longitudinal studies and analyses of other covariates so that a definite conclusion may be drawn.

## Figures and Tables

**Table 1:** Demographic, clinical, immunologic, and virologic characteristics of the patients studied.

Patient	Gender	Basal CD4^+^ (cells/mm^3^)	HCV VL range (log_10_ UI/mL)^†^	Fibrosis grade (METAVIR score)	Response to IFN-based therapy	Sampling times^*∗*^
A	F	U	<2.78–6.5	0	R	0, 24 h, 4 wk, 12 wk, 24 wk, 48 wk, 72 wk
B	F	644	4.5–6.7	4	NR	0, 24 h, 4 wk, 12 wk, 24 wk
C	M	304	5.3–6.8	2	NR	0, 24 h, 4 wk, 12 wk, 24 wk
E	M	957	<2.78–6.1	3	NR	0, 24 h, 3 wk, 12 wk, 24 wk
G	M	376	<2.78–6.8	2	NR	0, 24 h, 4 wk, 12 wk, 24 wk, 48 wk, 72 wk
H	M	130	5.3–7.1	0	NR	0, 24 h, 4 wk, 24 wk
J	M	817	3.6–7.1	1	NR	0, 24 h, 4 wk, 12 wk, 24 wk
K	M	400	6.4–6.7	4	NR	0, 24 h, 4 wk, 12 wk
L	M	625	5.9–7.4	3	NR	0, 24 h, 4 wk, 12 wk
M	M	414	4.2–6.3	3	NR	0, 24 h, 4 wk, 12 wk, 24 wk
N	M	303	6.0–6.6	2	NR	0, 24 h, 4 wk, 12 wk, 24 wk
P	M	622	<2.78–7.0	1	NR	0, 24 h, 4 wk, 12 wk, 24 wk
Q	M	468	<2.78–6.2	4	NR	0, 24 h, 4 wk, 12 wk, 24 wk, 48 wk, 72 wk
R	M	U	<2.78–7.5	0.5	R	0, 24 h, 4 wk, 12 wk, 24 wk, 48 wk, 72 wk
S	M	262	5.8–7.2	1	NR	0, 24 h, 4 wk, 12 wk, 24 wk
U	M	842	<2.78–6.7	4	R	0, 24 h, 4 wk, 12 wk, 24 wk, 48 wk
X	M	483	<2.78–6.1	1	NR	0, 24 h, 4 wk, 12 wk, 24 wk
Z	M	U	<2.78	4	R	0, 24 h, 4 wk, 12 wk, 24 wk, 48 wk, 72 wk

^*∗*^From basal; U: unknown; ^†^minimum and maximum values measured during the follow-up.

**Table 2:** Integrative selection analysis for HCV-core. NS: not significant.

Patient	Codon	SLAC	FEL	MEME
		*p* value	*p* value	*p* value
C	169	NS	0.04	NS
H	75	NS	0.03	0.03

## Additional Points

*Availability of Data and Material*. GenBank accession numbers for HCV core nucleotide sequences are MF622203 to MF622537. The datasets used and/or analyzed during the current study are available from the corresponding author on reasonable request.
